# Recurrence 14 Years After Initial Treatment of Extranodal NK/T Cell Lymphoma, Nasal Type

**DOI:** 10.7759/cureus.24177

**Published:** 2022-04-16

**Authors:** Ryo Kawaura, Masami Ohnishi, Tomoya Hori

**Affiliations:** 1 Department of Head and Neck Surgery-Otolaryngology, Ogaki Municipal Hospital, Ogaki, JPN

**Keywords:** devic, recurrence, nasal type, extranodal nk/t cell lymphoma, enkl

## Abstract

Extranodal NK/T cell lymphoma, nasal type (ENKL) primarily involves the nasal cavity. Although patients might visit an otorhinolaryngologist with nasal symptoms, such as nasal obstruction and epistaxis, it would be difficult to make a diagnosis correctly. We present a case of ENKL in which the patient had been in remission after initial treatment and relapsed 14 years after treatment. The patient had a worsening of nasal symptoms before the recurrence, but on this occasion, treatment such as sinusitis was successful in alleviating the symptoms. Although recurrence of lymphoma 10 years after treatment is rare, the possibility of recurrence should always be considered in post-malignant lymphoma cases as with any malignant tumor.

## Introduction

In the World Health Organization (WHO) Classification (2017), extranodal NK/T cell lymphoma, nasal type (ENKL) is classified as an NK (natural killer) cell tumor along with aggressive NK-cell leukemia and chronic lymphoproliferative disorder of NK cells [1]. Although the majority of ENKL cases originate from NK cells, it is often difficult to differentiate NK cells from T cells with current techniques. Therefore, both are collectively described as NK/T-cell lymphoma [2]. Because of the low efficacy of CHOP (cyclophosphamide, doxorubicin hydrochloride, oncovin, prednisolone) therapy and the rarity of the disease in ENKL, there is no standard treatment established by randomized controlled trials, and the recommended treatment differs among countries.

We report the case of an elderly gentleman who relapsed 14 years after initial treatment with RT-DeVIC (dexamethasone, carboplatin, etoposide, ifosfamide) therapy.

## Case presentation

At age 67, the patient visited our otolaryngology department for a tumor in the nasal cavity. Biopsy revealed a diagnosis of extranodal NK/T cell lymphoma, nasal type (ENKL), and he was referred to the department of hematology. After three courses of the DeVIC regimen (carboplatin, 300 mg/m^2^ on day 1; etoposide, 100 mg/m^2^ on days 1-3; ifosfamide, 1500 mg/m^2^ on Days 1-3; dexamethasone, 40 mg/body on Days 1-3) and radiation therapy (total 45Gy) as ENKL with clinical stage IB and the International Prognostic Index (IPI) 2 (LDH, age), the patient was followed up for 10 years, remained in remission, and completed regular visits to the hospital.

At the age of 79, he had nasal symptoms, such as increased purulent nasal discharge, and was referred to our otolaryngology department from his general practitioner's otolaryngology department. Although ENKL recurrence was suspected, his symptoms improved with antibiotic treatment and nasal rinsing.

**Figure 1:**
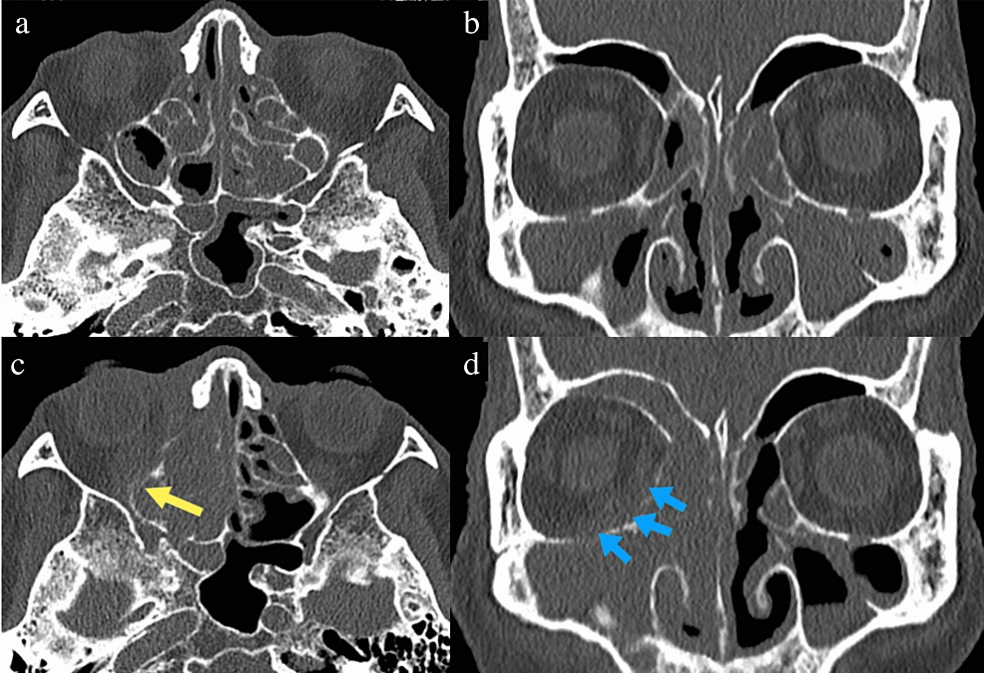
Images of computed tomography at the ages of 79 and 81 At age 81, compared with the computed tomography at age 79 (a-b), the intraorbital bony wall is obscured (c, yellow arrow). There is also an extension of the soft shadow within the orbit (d, blue arrow).

He was treated again at a general practitioner's otolaryngology clinic after more than a year; however, at age 81, he was referred to the otolaryngology clinic again because of a feeling of nasal obstruction and increased purulent nasal discharge from the right naris. He was treated with antibiotics but without any improvement (Figure 1). He had symptoms of oculomotor nerve palsy, such as diplopia and ptosis; thus, he underwent emergency endoscopic sinus surgery to decompress the orbit (Video 1). When the maxillary and ethmoid sinuses were opened, pus was drained, and the nasal mucosa was edematous and friable. Pathological diagnosis of the tissue obtained at that time suggested ENKL recurrence (Table 1), and the patient was referred to the hematology department for a second visit.

**Video 1:** Endoscopic surgery The intranasal tissue was very hemorrhagic. After collecting the tissue for pathology, surgical suction resection was performed with a device to decompress the intranasal and orbital areas.

**Table 1:** Pathological data of first and second visits At age 67 (first visit), LMP-1 was not positive but the diagnosis was ENKL. Although the details are unclear since it was more than a decade ago, we assume that the diagnosis was made because the histopathological findings and cellular markers were consistent with ENKL [3], and blood test results showed a history of infection with Epstein-Barr virus. LCA: Leukocyte Common Antigen; UCHL-1: Ubiquitin Carboxy-Terminal Hydrolase L1; CD: Cluster of Differentiation; Bcl: B-cell/CLL lymphoma 2; LMP-1: Latent membrane protein 1

	First visit (at age 67)	Second visit (at age 81)
morphological features	Invasive infiltrate of medium-sized lymphocytes with irregular nuclear margins, Absence of lymph follicular structure, No granuloma was formed.	Diffuse proliferation of atypical lymphocytes, Mitosis of lymphocytes, Presence of necrosis.
positive components	LCA, UCHL-1, CD3, Granzyme B, CD56, Bcl-2	CD3, Granzyme B, CD56, LMP-1
negative components	L26 (CD20), LMP-1	AE1/3, S100, CD20, CD30

Prior treatment with prednisolone (0.5 mg/kg/day) was initiated on the day after the second visit. The patient was discharged from the hospital, and a treatment plan was discussed; however, 15 days after the return visit, the patient was rushed to the emergency room with seizures and impaired consciousness. CT (computed tomography) and MRI (magnetic resonance imaging) of the head showed no obvious intracranial lesions, and CSF (cerebrospinal fluid) examination showed significant lymphocyte hyperplasia, which led to suspicion of CNS (central nervous system) infiltration. The patient was admitted to the hematology department. On Day 4 of hospitalization (18 days after the second visit), MTX (methotrexate; 15 mg), Ara-C (cytarabine; 40 mg), and DEX (dexamethasone, 4 mg) intrathecal infusion (IT) was administered, and symptoms of consciousness tended to improve. Because of his advanced age, SMILE (dexamethasone, methotrexate, ifosfamide, L-asparaginase, etoposide) therapy was avoided, and a reduction in the dose of DeVIC (50% dose, from the typical 2/3 to 3/4 dose), IT, and re-irradiation were planned.

After discharge from the hospital, he was again admitted to the scheduled hospital 46 days after the second visit, but his condition had deteriorated and his disease had progressed, with the appearance of elevated liver enzymes, increased lactate dehydrogenase (LDH) based on IPI, and new lesions in the adrenal glands on a whole-body CT scan. The DeVIC regimen was started the next day, and IT was performed again two days later; however, irradiation was discontinued because local irradiation was considered to be of little significance in an already advanced stage of the disease. Although LDH decreased with treatment, the patient required blood transfusions due to myelosuppression, his performance status also declined, and the future course of action was reconsidered. Then, 57 days after the second visit, the patient's oxygenation began to deteriorate, and in the early morning of 59 days after the second visit, the patient died. A pathological autopsy was proposed to investigate the cause; however, the bereaved family did not wish to have it.

## Discussion

Extranodal natural killer T-cell lymphoma, nasal type (ENKL) is a type of malignant lymphoma [1]. Epidemiologically, there are large racial differences in its incidence, and it is rare in Caucasians from Europe and the United States; however, it is not uncommon in Asians and Latin Americans [4]. Reports from South Korea and Hong Kong found an incidence of 7-8% of all malignant lymphomas [4]. The initial symptoms are often localized, such as nasal obstruction, rhinorrhea, and epistaxis, and as the disease progresses, tumor growth increases with bone destruction in the orbit, skull base, and palate [5]. In the advanced stage, tumor cells express multidrug-resistant P-glycoprotein that can extrude drugs, and the prognosis is poor regardless of the primary site [6-7].

In the case of a lesion in the nasal cavity that develops while destroying the surrounding area, ENKL is the differential, along with polyangiitis granulomatosa (conventional Wegener's granulomatosis) and other diseases. Multiple tissue biopsies may be required because of necrosis and hemorrhage, and immunohistochemistry may be helpful, along with EBER-1 *in situ* to confirm the presence of EBV (Epstein-Barr virus) [8].

Relapse of malignant lymphoma more than 10 years after remission is uncommon. Although scattered reports of relapses several years after remission in ENKL have been found, no reports of relapses more than 10 years after remission were identified. In this case, the patient had been treated for sinusitis before the recurrence. Even with this history, the possibility of recurrence must be considered when there is a history of lymphoma. Wang et al. reported that positive plasma EBV-DNA can predict ENKL recurrence and poor prognosis after asparaginase-based chemotherapy [9]. Although the initial treatment regimen should be considered, it may be worthwhile to consider searching for EBV-DNA and tissue biopsy when ENKL recurrence is suspected.

ENKL is a rare lymphoma and was once considered refractory. Although treatment varies from country to country because of epidemiological racial differences, in Japan, RT-2/3DeVIC, concurrent chemoradiotherapy, is recommended for first-onset localized ENKL, and SMILE therapy (dexamethasone, methotrexate, ifosfamide, L-asparaginase, etoposide), multidrug chemotherapy, is recommended for first-onset advanced stage and first relapse/refractory ENKL [2]. In this case, the initial onset was over a decade ago, and the dosage of the DeVIC regimen was different from the current recommendations. Additionally, at the time of recurrence, the patient was 81 years old, which was above the study age range (15-69 years) [10], and his general condition was not good; thus, the introduction of SMILE therapy was not recommended.

## Conclusions

In this case, we report a relapse 14 years after initial treatment with ENKL, which had previously been in remission. The patient developed worsening nasal symptoms before the recurrence, but at that time, the patient was successfully treated as a case of sinusitis. Clinicians should maintain a high index of suspicion for recurrence even in remote histories of ENKL in post-lymphoma treatment cases.
